# Supplementary material for: Hypoxic pregnancy promotes fibrosis and increases stress metabolites in the ovine fetal liver
Source: J Physiol. 2025 May 5;603(10):3223–43. doi: 10.1113/JP288724 (PMC12126611; doi:10.1113/JP288724)
Supplement: Supplementary file 2 — Table S1. Table S2. Table S3. Table S4. Table S5. [file TJP-603-3223-s001.docx]

**SUPPLEMENTARY MATERIALS**

**Table S1**. List of metabolites ranked by variable in projection (VIP) score.

| Metabolite | Compound ID | General category | VIP rank | VIP score |
| --- | --- | --- | --- | --- |
| Ectoine | C06231 | Inositol | 1 | 2.190 |
| acyl-C20:4 | ac125 | Carnitine and fatty acid metabolism | 2 | 2.061 |
| Hexadecenoyl-carnitine (acyl-C16:1) | HMDB06317 | Carnitine and fatty acid metabolism | 3 | 2.055 |
| O-octadecenoyl-L-carnitine (acyl-C18:1) | HMDB06351 | Carnitine and fatty acid metabolism | 4 | 1.939 |
| Indole-3-acetate | C00954 | Indole and Tryptophan | 5 | 1.874 |
| L-valine | C00183 | Amino acids | 6 | 1.863 |
| propionyl-carnitine (acyl-C3) | C03017 | Carnitine and fatty acid metabolism | 7 | 1.833 |
| gamma-Glutamyl-Se-methylselenocysteine | C05695 | Gamma-glutamyls | 8 | 1.819 |
| D-Glucono-1-5-lactone 6-phosphate | C01236 | Pentose Phosphate Pathway | 9 | 1.798 |
| GMP | C00144 | Nucleotides | 10 | 1.777 |
| AMP | C00020 | Nucleotides | 11 | 1.763 |
| 6-Hydroxykynurenic acid | C08480 | Indole and Tryptophan | 12 | 1.757 |
| Sphingosine | C00319 | Glycerophospholipid biosynthesis | 13 | 1.746 |
| acyl-C18:2 (Linoleoyl-CoA) | HMDB01064 | Carnitine and fatty acid metabolism | 14 | 1.739 |
| Sedoheptulose 1-phosphate | C06222 | Pentose Phosphate Pathway | 15 | 1.730 |
| D-Glucose 6-phosphate | C02965 | Glycolysis | 16 | 1.722 |
| UMP | C00105 | Nucleotides | 17 | 1.704 |
| D-4--Phosphopantothenate | C03492 | Panthothenate metabolism | 18 | 1.703 |
| D-Erythrose 4-phosphate | C00279 | Pentose Phosphate Pathway | 19 | 1.676 |
| acyl-C5:1 | HMDB02366 | Carnitine and fatty acid metabolism | 20 | 1.649 |
| acyl-C4-OH | HMDB13127 | Carnitine and fatty acid metabolism | 21 | 1.637 |
| D-Fructose 1-6-bisphosphate | C00354 | Glycolysis | 22 | 1.627 |
| D-Glyceraldehyde 3-phosphate/Glycerone phosphate | C00118 | Glycolysis | 23 | 1.621 |
| Biliverdin | C00500 | Hemolysis markers | 24 | 1.606 |
| Sphingosine 1-phosphate | C06124 | Glycerophospholipid biosynthesis | 25 | 1.589 |
| D-Rhamnose | C01684 | Other sugars | 26 | 1.581 |
| L-serine | C00065 | Amino acids | 27 | 1.574 |
| S-Adenosyl-L-methionine | C00019 | Serine biosynthesis and one-carbon metabolism | 28 | 1.572 |
| Ethanolamine phosphate | C00346 | Glycerophospholipid biosynthesis | 29 | 1.566 |
| 2-Hydroxyglutarate/Citramalate | C02630 | Alternative Carboxylic acids | 30 | 1.497 |
| L-phenylalanine | C00079 | Amino acids | 31 | 1.494 |
| L-glutamate | C00025 | Amino acids | 32 | 1.470 |
| Anthranilate | C00108 | Indole and Tryptophan | 33 | 1.460 |
| glycine | C00037 | Amino acids | 34 | 1.457 |
| Adenylosuccinic acid | C03794 | Nucleotides | 35 | 1.443 |
| 4-Acetamidobutanoate | C02946 | Arginine and proline metabolism | 36 | 1.443 |
| 5-Oxoproline | C01879 | GSH homeostasis | 37 | 1.442 |
| O-tetradecanoyl-L-carnitine (acyl-C14) | HMDB05066 | Carnitine and fatty acid metabolism | 38 | 1.380 |
| L-Palmitoylcarnitine (acyl-C16) | C02990 | Carnitine and fatty acid metabolism | 39 | 1.372 |
| 2/3-Phospho-D-glycerate | C00631 | Glycolysis | 40 | 1.361 |
| Cytidine | C00475 | Nucleotides | 41 | 1.359 |
| Octadecanoyl-L-carnitine (acyl-C18) | HMDB00848 | Carnitine and fatty acid metabolism | 42 | 1.355 |
| Phosphoenolpyruvate | C00074 | Glycolysis | 43 | 1.332 |
| 10-Formyltetrahydrofolate | C00234 | Folate pool (One carbon metabolism) | 44 | 1.315 |
| D-Glucose | C00031 | Glycolysis | 45 | 1.312 |
| Dimethylglycine | C01026 | Serine biosynthesis and one-carbon metabolism | 46 | 1.303 |
| quinolinic acid | C03722 | Indole and Tryptophan | 47 | 1.287 |
| S-Adenosyl-L-homocysteine | C00021 | Serine biosynthesis and one-carbon metabolism | 48 | 1.278 |
| gamma-L-Glutamylputrescine | C15699 | Gamma-glutamyls | 49 | 1.218 |
| L-Adrenaline | C00788 | Signaling | 50 | 1.200 |
| gamma-L-Glutamyl-L-cysteine | C00669 | Gamma-glutamyls | 51 | 1.186 |
| ADP-D-ribose | C01882 | Nucleotides | 52 | 1.181 |
| L-alanine | C00041 | Amino acids | 53 | 1.174 |
| gamma-Glutamyl-gamma-aminobutyrate | C15767 | Gamma-glutamyls | 54 | 1.152 |
| Malate | C00149 | TCA cycle | 55 | 1.150 |
| Pyridoxal | C00250 | Nucleotides | 56 | 1.131 |
| Citrate | C00158 | TCA cycle | 57 | 1.129 |
| L-Methionine S-oxide | C02989 | Sulfur metabolism | 58 | 1.113 |
| Urate | C00366 | Nucleotides | 59 | 1.069 |
| Adenosine | C00212 | Nucleotides | 60 | 1.065 |
| L-Citrulline | C00327 | Urea cycle | 61 | 1.062 |
| Cystathionine | C00542 | Serine biosynthesis and one-carbon metabolism | 62 | 1.056 |
| Bilirubin | C00486 | Hemolysis markers | 63 | 1.054 |
| L-leucine/isoleucine | C00123 | Amino acids | 64 | 1.053 |
| L-lysine | C00047 | Amino acids | 65 | 1.050 |
| Diphosphate | C00013 | Phosphates | 66 | 1.046 |
| D-Ribose | C00121 | Other sugars | 67 | 1.046 |
| a-Linolenic acid (Octadecatrienoic acid) | C06427 | Poly-unsaturated Fatty Acids | 68 | 1.045 |
| 5-Hydroxyisourate | C11821 | Nucleotides | 69 | 1.039 |
| L-proline | C00148 | Amino acids | 70 | 1.038 |
| IMP | C00130 | Nucleotides | 71 | 1.031 |
| 6-Phospho-D-gluconate | C00345 | Pentose Phosphate Pathway | 72 | 1.030 |
| NADP+ | C00006 | Nucleotides | 73 | 1.025 |
| Ascorbate | C00072 | GSH homeostasis | 74 | 1.014 |
| 1-4-beta-D-Xylan | C02352 | Aminosugars | 75 | 1.012 |
| ITP | C00081 | Nucleotides | 76 | 1.010 |
| GDP | C00035 | Nucleotides | 77 | 0.981 |
| Pyridoxamine 5'-phosphate | C00647 | Nucleotides | 78 | 0.980 |
| Phosphoserine | C02532 | Serine biosynthesis and one-carbon metabolism | 79 | 0.970 |
| Xanthine | C00385 | Nucleotides | 80 | 0.958 |
| D-Glucosamine | C00329 | Aminosugars | 81 | 0.932 |
| Lactate | C01432 | Glycolysis | 82 | 0.928 |
| O-dodecanoyl-carnitine (acyl-C12) | HMDB02250 | Carnitine and fatty acid metabolism | 83 | 0.927 |
| 2-Oxoglutaramate | C00940 | TCA cycle | 84 | 0.918 |
| 3-Sulfocatechol | C06336 | Sulfur metabolism | 85 | 0.918 |
| 2-Aminomuconate | C02220 | Indole and Tryptophan | 86 | 0.892 |
| Choline | C00114 | Glycerophospholipid biosynthesis | 87 | 0.887 |
| Docosahexaenoic acid | C06429 | Poly-unsaturated Fatty Acids | 88 | 0.881 |
| 5-Hydroxyindoleacetate | C05635 | Indole and Tryptophan | 89 | 0.879 |
| L-methionine | C00073 | Amino acids | 90 | 0.871 |
| N-Glycoloyl-neuraminate | C03410 | Aminosugars | 91 | 0.868 |
| Pantothenate | C00864 | Panthothenate metabolism | 92 | 0.865 |
| L-tyrosine | C00082 | Amino acids | 93 | 0.852 |
| gamma-L-Glutamyl-D-alanine | C03738 | Gamma-glutamyls | 94 | 0.831 |
| L-tryptophan | C00078 | Amino acids | 95 | 0.831 |
| (7Z-10Z-13Z-16Z-19Z)-Docosa-7-10-13-16-19-pentaenoic acid | C16513 | Essential fatty acids | 96 | 0.826 |
| Creatinine | C00791 | Arginine and proline metabolism | 97 | 0.794 |
| Spermine | C00750 | Polyamines | 98 | 0.779 |
| L-glutamine | C00064 | Amino acids | 99 | 0.778 |
| 5-6-Dihydrothymine | C00906 | Nucleotides | 100 | 0.767 |
| Octadecenoic acid (Oleic acid) | C00712 | Monounsaturated Fatty Acids | 101 | 0.767 |
| Phosphate | C00009 | Phosphates | 102 | 0.761 |
| L-Carnitine | C00318 | Carnitine and fatty acid metabolism | 103 | 0.753 |
| (8Z-11Z-14Z)-Icosatrienoic acid | C03242 | Essential fatty acids | 104 | 0.748 |
| Taurine | C00245 | Sulfur metabolism | 105 | 0.747 |
| (R)-S-Lactoylglutathione | C03451 | Sulfur metabolism | 106 | 0.739 |
| Fumarate | C00122 | TCA cycle | 107 | 0.737 |
| Argininosuccinate | C03406 | Urea cycle | 108 | 0.731 |
| Arachidonic acid (Eicosatetraenoic acid) | C00219 | Poly-unsaturated Fatty Acids | 109 | 0.705 |
| N-Acetylneuraminate | C00270 | Aminosugars | 110 | 0.703 |
| Cys-Gly | C01419 | GSH homeostasis | 111 | 0.677 |
| alpha-D-Ribose 1-phosphate | C00620 | Pentose Phosphate Pathway | 112 | 0.652 |
| Cytosine | C00380 | Nucleotides | 113 | 0.644 |
| alpha-D-Glucosamine 1-phosphate | C06156 | Aminosugars | 114 | 0.638 |
| UDP | C00015 | Nucleotides | 115 | 0.625 |
| Dopamine | C03758 | Signaling | 116 | 0.624 |
| Eicosapentaenoic acid | C06428 | Poly-unsaturated Fatty Acids | 117 | 0.620 |
| Uracil | C00106 | Nucleotides | 118 | 0.615 |
| 3-Sulfino-L-alanine | C00606 | Sulfur metabolism | 119 | 0.606 |
| Indole-3-acetaldehyde | C00637 | Indole and Tryptophan | 120 | 0.603 |
| CMP | C00055 | Nucleotides | 121 | 0.573 |
| 5-Phosphoribosylamine | C03090 | Nucleotides | 122 | 0.567 |
| 4-Pyridoxate | C00847 | Nucleotides | 123 | 0.563 |
| ADP | C00008 | Nucleotides | 124 | 0.562 |
| Adenine | C00147 | Nucleotides | 125 | 0.555 |
| L-threonine | C00188 | Amino acids | 126 | 0.554 |
| Serotonin | C00780 | Signaling | 127 | 0.552 |
| Pantetheine 4--phosphate | C01134 | Panthothenate metabolism | 128 | 0.547 |
| NADH | C00004 | Nucleotides | 129 | 0.530 |
| kynurenine | C00328 | Indole and Tryptophan | 130 | 0.511 |
| L-aspartate | C00049 | Amino acids | 131 | 0.508 |
| L-asparagine | C00152 | Amino acids | 132 | 0.501 |
| dAMP | C00360 | Nucleotides | 133 | 0.498 |
| Allantoate | C00499 | Nucleotides | 134 | 0.498 |
| UTP | C00075 | Nucleotides | 135 | 0.496 |
| Hypoxanthine | C00262 | Nucleotides | 136 | 0.495 |
| Creatine | C00300 | Arginine and proline metabolism | 137 | 0.491 |
| Glutathione disulfide | C00127 | GSH homeostasis | 138 | 0.484 |
| Hypotaurine | C00519 | Sulfur metabolism | 139 | 0.479 |
| Putrescine | C00134 | Polyamines | 140 | 0.469 |
| NADPH | C00005 | Nucleotides | 141 | 0.464 |
| Hexadecenoic acid (Palmitoleic acid) | C08362 | Monounsaturated Fatty Acids | 142 | 0.443 |
| Inosine | C00294 | Nucleotides | 143 | 0.442 |
| Glycerol 3-phosphate | C00093 | Glycerophospholipid biosynthesis | 144 | 0.438 |
| Pyruvate | C00022 | Glycolysis | 145 | 0.428 |
| Nonanoic acid (pelargonate) | C01601 | Saturated Fatty acids | 146 | 0.428 |
| Carnosine | C00386 | Arginine and proline metabolism | 147 | 0.427 |
| L-cysteine | C00097 | Amino acids | 148 | 0.409 |
| Guanosine | C00387 | Nucleotides | 149 | 0.403 |
| L-octanoylcarnitine (acyl-C8) | HMDB00791 | Carnitine and fatty acid metabolism | 150 | 0.398 |
| L-Homocysteine | C00155 | Serine biosynthesis and one-carbon metabolism | 151 | 0.398 |
| L-cystine | C00491 | Amino acids | 152 | 0.362 |
| hexanoyl-L-carnitine (acyl-C6) | HMDB00756 | Carnitine and fatty acid metabolism | 153 | 0.354 |
| N-Methylethanolamine phosphate | C01210 | Glycerophospholipid biosynthesis | 154 | 0.353 |
| NAD+ | C00003 | Nucleotides | 155 | 0.340 |
| acetyl-carnitine (acyl-C2) | C02571 | Carnitine and fatty acid metabolism | 156 | 0.335 |
| D-Arabitol | C01904 | Other sugars | 157 | 0.333 |
| Octanoic acid (caprylate) | C06423 | Saturated Fatty acids | 158 | 0.313 |
| S-Glutathionyl-L-cysteine | C05526 | GSH homeostasis | 159 | 0.299 |
| (5-L-Glutamyl)-L-glutamine | C05283 | Gamma-glutamyls | 160 | 0.290 |
| acyl-C5-OH | ac107 | Carnitine and fatty acid metabolism | 161 | 0.288 |
| Hexadecanoic acid (palmitic acid) | C00249 | Saturated Fatty acids | 162 | 0.285 |
| CMP-N-acetylneuraminate | C00128 | Aminosugars | 163 | 0.271 |
| UDP-N-acetyl-D-glucosamine | C00043 | Aminosugars | 164 | 0.266 |
| UDP-glucose | C00029 | Nucleotides | 165 | 0.264 |
| Linoleic acid ((9Z,12Z)-Octadecadienoic acid) | C01595 | Poly-unsaturated Fatty Acids | 166 | 0.262 |
| butanoyl-l-carnitine (acyl-C4) | C02862 | Carnitine and fatty acid metabolism | 167 | 0.255 |
| Heptanoic acid | C17714 | Saturated Fatty acids | 168 | 0.253 |
| Phosphocreatine | C02305 | Arginine and proline metabolism | 169 | 0.245 |
| Decanoic acid (caprate) | C01571 | Saturated Fatty acids | 170 | 0.245 |
| Flavin mononucleotide | C00061 | Nucleotides | 171 | 0.230 |
| Dehydroascorbate | C05422 | GSH homeostasis | 172 | 0.196 |
| acyl-C4-DC | HMDB13133 | Carnitine and fatty acid metabolism | 173 | 0.193 |
| Glutathione | C00051 | GSH homeostasis | 174 | 0.191 |
| Octadecanoic acid (stearic acid) | C01530 | Saturated Fatty acids | 175 | 0.174 |
| Spermidine | C00315 | Polyamines | 176 | 0.173 |
| Acetylcholine | C01996 | Glycerophospholipid biosynthesis | 177 | 0.168 |
| Ornithine | C01602 | Urea cycle | 178 | 0.168 |
| Guanine | C00242 | Nucleotides | 179 | 0.157 |
| acyl-C20 | ac124 | Carnitine and fatty acid metabolism | 180 | 0.155 |
| Dodecanoic acid (lauric acid) | C02679 | Saturated Fatty acids | 181 | 0.142 |
| N-Acetylornithine | C00437 | Arginine and proline metabolism | 182 | 0.132 |
| L-histidine | C00135 | Amino acids | 183 | 0.125 |
| 2-Oxoglutarate | C00026 | TCA cycle | 184 | 0.125 |
| ATP | C00002 | Nucleotides | 185 | 0.110 |
| Succinate | C00042 | TCA cycle | 186 | 0.087 |
| Tetradecenoyl Carnitine (acyl-C14:1) | HMDB13329 | Carnitine and fatty acid metabolism | 187 | 0.065 |
| Homocarnosine | C00884 | Arginine and proline metabolism | 188 | 0.061 |
| Tetradecenoic acid (myristoleic acid) | C08322 | Monounsaturated Fatty Acids | 189 | 0.059 |
| 3',5'-Cyclic AMP | C00575 | Nucleotides | 190 | 0.055 |
| (S)(+)-Allantoin | C02350 | Nucleotides | 191 | 0.030 |
| Mannitol | C00392 | Other sugars | 192 | 0.028 |
| Nicotinate ribonucleotide | C01185 | Nucleotides | 193 | 0.025 |
| Tetradecanoic acid (myristic acid) | C06424 | Saturated Fatty acids | 194 | 0.018 |
| 5-10-Methenyltetrahydrofolate | C00445 | Folate pool (One carbon metabolism) | 195 | 0.012 |
| trans-4-Hydroxy-L-proline | C01157 | Arginine and proline metabolism | 196 | 0.003 |
| Nicotinamide | C00153 | Nucleotides | 197 | 0.003 |

**Table S2.** Differentially expressed genes.

| Ensemble ID | Gene symbol | Log_2_Fold Change | Q value |
| --- | --- | --- | --- |
| ENSOARG00020023447 | *GPD1* | -1.239 | <0.001 |
| ENSOARG00020000805 | *DNAJC22* | -1.343 | <0.001 |
| ENSOARG00020003193 | *GSS* | -0.746 | <0.001 |
| ENSOARG00020006108 | *TAGLN3* | -1.034 | <0.001 |
| ENSOARG00020010401 |  | -1.140 | <0.001 |
| ENSOARG00020014833 | *AOX1* | 1.052 | <0.001 |
| ENSOARG00020019964 | *FKBP1B* | -1.317 | <0.001 |
| ENSOARG00020010589 | *SPON2* | 0.898 | <0.001 |
| ENSOARG00020014217 |  | -0.829 | 0.00154 |
| ENSOARG00020025989 | *PPM1F* | 0.474 | 0.00179 |
| ENSOARG00020012973 |  | -0.659 | 0.00196 |
| ENSOARG00020018010 | *CALU* | -0.549 | 0.00361 |
| ENSOARG00020014334 | *ISG15* | -0.968 | 0.00473 |
| ENSOARG00020005712 | *PCYOX1* | -0.602 | 0.00583 |
| ENSOARG00020018696 | *S100A10* | -0.609 | 0.00583 |
| ENSOARG00020014533 | *CBR1* | -0.706 | 0.00801 |
| ENSOARG00020022539 |  | -0.662 | 0.00858 |
| ENSOARG00020025991 | *TUFM* | -0.313 | 0.00858 |
| ENSOARG00020014789 | *NHP2* | -0.599 | 0.00858 |
| ENSOARG00020002749 | *AFP* | -3.072 | 0.00858 |
| ENSOARG00020009943 | *LECT2* | -0.581 | 0.01092 |
| ENSOARG00020023529 | *MPI* | -0.558 | 0.01214 |
| ENSOARG00020019703 | *PRR13* | -0.543 | 0.01257 |
| ENSOARG00020007257 | *C4B* | -0.922 | 0.01444 |
| ENSOARG00020022141 |  | -0.436 | 0.01582 |
| ENSOARG00020018117 | *SASH1* | 0.612 | 0.01719 |
| ENSOARG00020023340 |  | -0.359 | 0.01719 |
| ENSOARG00020007954 | *OXER1* | -0.826 | 0.01814 |
| ENSOARG00020001820 | *DQX1* | -1.071 | 0.01814 |
| ENSOARG00020013409 | *S100B* | -1.297 | 0.01814 |
| ENSOARG00020012152 | *SLC39A7* | -0.308 | 0.01848 |
| ENSOARG00020011962 | *TMEM230* | -0.335 | 0.01848 |
| ENSOARG00020002356 | *PSAT1* | -0.751 | 0.01848 |
| ENSOARG00020002932 | *GSN* | -1.057 | 0.01848 |
| ENSOARG00020016397 | *ZHX2* | 0.559 | 0.01853 |
| ENSOARG00020008695 | *RPN2* | -0.263 | 0.01962 |
| ENSOARG00020014065 | *SGPL1* | -0.279 | 0.01962 |
| ENSOARG00020021252 | *IMP4* | -0.483 | 0.01969 |
| ENSOARG00020006110 | *EPSTI1* | -0.656 | 0.01969 |
| ENSOARG00020010357 | *GSTO1* | -0.463 | 0.02018 |
| ENSOARG00020011496 | *TBCB* | -0.412 | 0.02065 |
| ENSOARG00020015168 |  | -0.806 | 0.02180 |
| ENSOARG00020004359 | *THBS4* | 0.940 | 0.02204 |
| ENSOARG00020022321 |  | -0.888 | 0.02204 |
| ENSOARG00020001920 |  | -0.429 | 0.02204 |
| ENSOARG00020007309 | *XRN1* | 0.500 | 0.02204 |
| ENSOARG00020010734 | *FAM117A* | 0.474 | 0.02204 |
| ENSOARG00020021017 | *PNPO* | -0.497 | 0.02204 |
| ENSOARG00020012170 | *LGALS3BP* | -0.420 | 0.02246 |
| ENSOARG00020012211 | *UBE2L6* | -0.490 | 0.02246 |
| ENSOARG00020012751 | *MRPL13* | -0.397 | 0.02334 |
| ENSOARG00020004257 | *EAF2* | -0.489 | 0.02334 |
| ENSOARG00020023593 | *DDOST* | -0.400 | 0.02339 |
| ENSOARG00020003114 | *INTS6* | 0.267 | 0.02352 |
| ENSOARG00020002298 | *PBXIP1* | -0.397 | 0.02352 |
| ENSOARG00020000023 | *COX3* | -0.481 | 0.02352 |
| ENSOARG00020009354 | *TMED6* | -0.837 | 0.02565 |
| ENSOARG00020014797 | *GLYAT* | -1.027 | 0.02565 |
| ENSOARG00020011634 | *CALML4* | -0.704 | 0.02713 |
| ENSOARG00020022143 | *MRPS23* | -0.324 | 0.02733 |
| ENSOARG00020023750 | *ADK* | -0.559 | 0.03024 |
| ENSOARG00020000990 | *AIF1L* | -0.748 | 0.03024 |
| ENSOARG00020021248 | *CD34* | 1.098 | 0.03066 |
| ENSOARG00020006773 | *DNAJC4* | -0.543 | 0.03066 |
| ENSOARG00020021341 | *SLC22A7* | -0.586 | 0.03066 |
| ENSOARG00020024009 | *STX2* | 0.719 | 0.03169 |
| ENSOARG00020019239 | *ARHGAP23* | 0.654 | 0.03169 |
| ENSOARG00020019211 | *HGFAC* | 0.487 | 0.03169 |
| ENSOARG00020021065 | *TMEM167A* | -0.459 | 0.03169 |
| ENSOARG00020020704 | *FKBP7* | -0.515 | 0.03169 |
| ENSOARG00020018153 | *CYB5RL* | -0.554 | 0.03169 |
| ENSOARG00020005458 | *RCAN1* | -1.118 | 0.03169 |
| ENSOARG00020015085 | *MXI1* | 0.686 | 0.03278 |
| ENSOARG00020001749 |  | -0.932 | 0.03278 |
| ENSOARG00020009127 | *EIF4B* | 0.266 | 0.03278 |
| ENSOARG00020016513 | *MRPL16* | -0.346 | 0.03278 |
| ENSOARG00020015235 | *DPM2* | -0.553 | 0.03278 |
| ENSOARG00020021515 | *CRIP3* | -0.569 | 0.03278 |
| ENSOARG00020012804 | *FBXO21* | -0.346 | 0.03556 |
| ENSOARG00020017442 | *ZEB1* | 0.395 | 0.03571 |
| ENSOARG00020000869 | *UBL5* | -0.297 | 0.03571 |
| ENSOARG00020024699 | *CLDN15* | -0.475 | 0.03571 |
| ENSOARG00020025412 | *NOTUM* | 0.747 | 0.03603 |
| ENSOARG00020004601 | *GALT* | -0.657 | 0.03611 |
| ENSOARG00020011347 | *SUN2* | 0.259 | 0.03675 |
| ENSOARG00020023500 | *ZNF646* | 0.304 | 0.03732 |
| ENSOARG00020012843 | *PLAGL1* | 1.585 | 0.03852 |
| ENSOARG00020005902 | *SELENOF* | -0.313 | 0.03854 |
| ENSOARG00020015494 | *PPP1R1A* | -0.669 | 0.03943 |
| ENSOARG00020001072 | *PLD1* | -0.491 | 0.04131 |
| ENSOARG00020021450 | *TMEM255A* | -0.827 | 0.04167 |
| ENSOARG00020021555 |  | -1.050 | 0.04180 |
| ENSOARG00020021015 | *IYD* | -0.674 | 0.04180 |
| ENSOARG00020016552 | *SIL1* | -0.310 | 0.04262 |
| ENSOARG00020002248 | *CHRD* | -0.431 | 0.04342 |
| ENSOARG00020010899 | *CPN2* | -0.371 | 0.04374 |
| ENSOARG00020019962 | *HOPX* | -1.138 | 0.04532 |
| ENSOARG00020004966 | *TUBGCP3* | 0.462 | 0.04557 |
| ENSOARG00020012878 | *TAB1* | 0.390 | 0.04571 |
| ENSOARG00020014228 | *PCBD1* | -0.343 | 0.04571 |
| ENSOARG00020000017 | *COX1* | -0.545 | 0.04571 |
| ENSOARG00020006875 | *KDELR2* | -0.269 | 0.04929 |
| ENSOARG00020007774 | *EMC1* | -0.289 | 0.04929 |
| ENSOARG00020008010 | *CDK18* | -0.326 | 0.04929 |
| ENSOARG00020024349 | *AGPAT1* | -0.361 | 0.04929 |
| ENSOARG00020010617 | *PSME1* | -0.384 | 0.04929 |
| ENSOARG00020024135 | *TMEM97* | -0.445 | 0.04929 |
| ENSOARG00020016734 | *POLA2* | 0.491 | 0.05017 |
| ENSOARG00020008562 | *ARPC5L* | -0.364 | 0.05017 |
| ENSOARG00020004798 | *SIGMAR1* | -0.480 | 0.05017 |
| ENSOARG00020011168 | *PITPNC1* | 0.541 | 0.05033 |
| ENSOARG00020019130 | *LRIG3* | 0.365 | 0.05033 |
| ENSOARG00020011202 | *DAD1* | -0.351 | 0.05033 |
| ENSOARG00020008837 | *RPS27L* | -0.411 | 0.05057 |
| ENSOARG00020001079 | *SSR3* | -0.419 | 0.05057 |
| ENSOARG00020010609 | *EEA1* | 0.493 | 0.05075 |
| ENSOARG00020010413 | *NDUFAF1* | -0.462 | 0.05183 |
| ENSOARG00020011081 | *NGEF* | -0.523 | 0.05280 |
| ENSOARG00020016713 | *CKB* | -0.663 | 0.05280 |
| ENSOARG00020012456 |  | -0.746 | 0.05394 |
| ENSOARG00020006915 | *TMED1* | -0.302 | 0.05394 |
| ENSOARG00020005652 | *DYNLT1* | -0.319 | 0.05394 |
| ENSOARG00020022358 | *NUDC* | -0.331 | 0.05394 |
| ENSOARG00020010267 | *DIRAS3* | -0.556 | 0.05394 |
| ENSOARG00020021403 | *VAT1* | 0.451 | 0.05622 |
| ENSOARG00020013976 |  | -0.391 | 0.05764 |
| ENSOARG00020009078 | *CHMP4A* | -0.292 | 0.05764 |
| ENSOARG00020017410 | *GALNT11* | -0.327 | 0.05764 |
| ENSOARG00020025423 | *OSTC* | -0.383 | 0.05764 |
| ENSOARG00020026108 | *GPATCH4* | -0.568 | 0.05764 |
| ENSOARG00020017764 | *PIGQ* | 0.706 | 0.05842 |
| ENSOARG00020002129 | *MGST1* | -0.600 | 0.05846 |
| ENSOARG00020014694 | *PSPH* | -0.867 | 0.05862 |
| ENSOARG00020025804 | *PEX26* | 0.328 | 0.05884 |
| ENSOARG00020000938 | *DUSP12* | -0.402 | 0.05884 |
| ENSOARG00020008908 | *C9* | -0.359 | 0.06021 |
| ENSOARG00020015620 | *CRCP* | -0.601 | 0.06021 |
| ENSOARG00020011596 | *MINDY1* | 0.543 | 0.06075 |
| ENSOARG00020001312 | *ASPN* | 0.407 | 0.06147 |
| ENSOARG00020023575 | *MESD* | -0.340 | 0.06179 |
| ENSOARG00020014611 | *GAA* | 0.455 | 0.06206 |
| ENSOARG00020001496 | *CRIM1* | 0.433 | 0.06206 |
| ENSOARG00020009104 | *ACAP2* | 0.425 | 0.06206 |
| ENSOARG00020001933 | *STAT6* | 0.394 | 0.06206 |
| ENSOARG00020013834 |  | 0.381 | 0.06206 |
| ENSOARG00020002218 | *STRAP* | -0.204 | 0.06206 |
| ENSOARG00020015600 | *SSR2* | -0.333 | 0.06206 |
| ENSOARG00020018493 | *LTO1* | -0.340 | 0.06206 |
| ENSOARG00020003074 | *TMEM150A* | -0.367 | 0.06206 |
| ENSOARG00020024475 | *GLT8D1* | -0.391 | 0.06206 |
| ENSOARG00020019735 | *BAX* | -0.404 | 0.06206 |
| ENSOARG00020012227 | *HSP10* | -0.447 | 0.06206 |
| ENSOARG00020025061 | *JAGN1* | -0.457 | 0.06206 |
| ENSOARG00020007901 | *HAAO* | -0.495 | 0.06206 |
| ENSOARG00020014531 | *TMED9* | -0.261 | 0.06234 |
| ENSOARG00020001504 |  | -0.492 | 0.06617 |
| ENSOARG00020017255 |  | 0.278 | 0.06617 |
| ENSOARG00020022894 | *TMED3* | -0.540 | 0.06617 |
| ENSOARG00020002724 | *GUCY2C* | -0.551 | 0.06617 |
| ENSOARG00020017879 | *CRYAB* | -0.709 | 0.06628 |
| ENSOARG00020016304 | *CACNB1* | 0.657 | 0.06772 |
| ENSOARG00020020068 | *RAP1GAP* | 0.863 | 0.06992 |
| ENSOARG00020000424 | *DNAAF10* | -0.417 | 0.07085 |
| ENSOARG00020021046 | *ASGR2* | -0.349 | 0.07125 |
| ENSOARG00020006059 | *SOD2* | -0.496 | 0.07150 |
| ENSOARG00020012177 | *MRAS* | -0.885 | 0.07150 |
| ENSOARG00020010836 | *CHID1* | -0.369 | 0.07427 |
| ENSOARG00020013278 | *RPS19BP1* | -0.408 | 0.07427 |
| ENSOARG00020013824 | *SCFD2* | -0.464 | 0.07427 |
| ENSOARG00020016542 | *B3GALNT2* | -0.339 | 0.07503 |
| ENSOARG00020000969 | *TSPO* | 0.942 | 0.07546 |
| ENSOARG00020019180 | *EHBP1L1* | 0.930 | 0.07546 |
| ENSOARG00020022305 | *SRA1* | -0.351 | 0.07546 |
| ENSOARG00020012828 | *NUP98* | 0.309 | 0.07718 |
| ENSOARG00020004956 | *GPSM2* | 1.042 | 0.07749 |
| ENSOARG00020025706 | *CHD7* | 0.329 | 0.07784 |
| ENSOARG00020000634 | *SPRED2* | 0.329 | 0.07795 |
| ENSOARG00020013754 | *SULT2A1* | 1.635 | 0.07811 |
| ENSOARG00020020191 | *SLC11A2* | 0.559 | 0.07811 |
| ENSOARG00020006446 |  | -0.928 | 0.07811 |
| ENSOARG00020018354 | *GUK1* | -0.254 | 0.07811 |
| ENSOARG00020008830 | *GTPBP4* | -0.445 | 0.07811 |
| ENSOARG00020018007 | *FAM114A2* | -0.297 | 0.07876 |
| ENSOARG00020020155 |  | -0.738 | 0.08065 |
| ENSOARG00020008002 | *ZDHHC24* | -0.458 | 0.08065 |
| ENSOARG00020017467 | *TNRC6C* | 0.418 | 0.08076 |
| ENSOARG00020012420 | *IGSF8* | 0.558 | 0.08303 |
| ENSOARG00020020002 | *SESTD1* | -0.321 | 0.08303 |
| ENSOARG00020001159 | *ASF1B* | 0.802 | 0.08365 |
| ENSOARG00020002583 | *TRIM24* | 0.420 | 0.08365 |
| ENSOARG00020023084 | *CEP350* | 0.360 | 0.08365 |
| ENSOARG00020011055 | *GLRX* | -0.478 | 0.08365 |
| ENSOARG00020008841 | *EMID1* | -0.485 | 0.08365 |
| ENSOARG00020002378 | *NCBP2* | -0.274 | 0.08385 |
| ENSOARG00020017339 | *EEFSEC* | 0.406 | 0.08408 |
| ENSOARG00020015437 | *KDM3B* | 0.299 | 0.08408 |
| ENSOARG00020016965 | *ZNF335* | 0.236 | 0.08408 |
| ENSOARG00020004525 |  | 2.184 | 0.08408 |
| ENSOARG00020003286 | *PTP4A1* | -0.348 | 0.08408 |
| ENSOARG00020000884 | *S100A16* | -0.351 | 0.08408 |
| ENSOARG00020000027 | *ND4* | -0.387 | 0.08408 |
| ENSOARG00020025329 | *AAMDC* | -0.424 | 0.08408 |
| ENSOARG00020003336 | *MMAB* | -0.441 | 0.08408 |
| ENSOARG00020011429 | *TICRR* | 0.806 | 0.08520 |
| ENSOARG00020024580 |  | -0.413 | 0.08520 |
| ENSOARG00020018837 |  | 0.314 | 0.08520 |
| ENSOARG00020006523 | *MRPL11* | -0.344 | 0.08588 |
| ENSOARG00020001722 | *SEC22B* | -0.327 | 0.08644 |
| ENSOARG00020008807 | *EPOR* | 1.069 | 0.08923 |
| ENSOARG00020018494 | *UFC1* | -0.294 | 0.08923 |
| ENSOARG00020004808 | *LSM4* | -0.358 | 0.08923 |
| ENSOARG00020005891 | *ZNF395* | 0.388 | 0.08948 |
| ENSOARG00020015472 |  | -0.352 | 0.08967 |
| ENSOARG00020001013 | *UMPS* | -0.358 | 0.09018 |
| ENSOARG00020016261 | *NECTIN1* | 0.473 | 0.09027 |
| ENSOARG00020021147 | *TM6SF1* | -0.381 | 0.09048 |
| ENSOARG00020017693 | *FADS3* | 0.999 | 0.09366 |
| ENSOARG00020021045 | *TRIM4* | 0.399 | 0.09460 |
| ENSOARG00020021798 | *TXN2* | -0.228 | 0.09460 |
| ENSOARG00020022953 | *COX15* | -0.335 | 0.09460 |
| ENSOARG00020016132 | *CLEC16A* | -0.355 | 0.09460 |
| ENSOARG00020014905 | *SRP19* | -0.425 | 0.09460 |
| ENSOARG00020023847 | *MCEE* | -0.380 | 0.09469 |
| ENSOARG00020001069 | *TIPARP* | -0.471 | 0.09595 |
| ENSOARG00020024535 | *COL26A1* | -0.729 | 0.09595 |
| ENSOARG00020003523 | *GCSH* | -0.447 | 0.09596 |
| ENSOARG00020013990 | *SHPRH* | 0.302 | 0.09670 |
| ENSOARG00020003553 | *NDUFA3* | -0.439 | 0.09670 |
| ENSOARG00020004646 | *NUP54* | -0.450 | 0.09670 |
| ENSOARG00020009863 | *UVSSA* | 0.356 | 0.09831 |
| ENSOARG00020002426 | *THPO* | -0.387 | 0.09831 |
| ENSOARG00020017245 | *ATOX1* | -0.427 | 0.09831 |
| ENSOARG00020024995 | *ANK1* | 0.749 | 0.09832 |
| ENSOARG00020014299 | *WNK1* | 0.394 | 0.09832 |
| ENSOARG00020022861 | *VPS25* | -0.329 | 0.09832 |
| ENSOARG00020003204 | *ZNF575* | -0.427 | 0.09832 |
| ENSOARG00020024385 | *EGFL8* | -0.693 | 0.09832 |
| ENSOARG00020010299 | *XYLB* | 0.610 | 0.09897 |
| ENSOARG00020012750 | *CD163* | -0.447 | 0.09897 |
| ENSOARG00020002539 | *HINT1* | 0.491 | 0.09904 |
| ENSOARG00020009523 | *HIGD2A* | -0.375 | 0.09904 |
| ENSOARG00020010500 | *MRPS22* | -0.309 | 0.10132 |
| ENSOARG00020026178 | *SPAG9* | 0.387 | 0.10134 |
| ENSOARG00020006189 | *F7* | -0.464 | 0.10134 |
| ENSOARG00020003757 |  | 0.286 | 0.10167 |
| ENSOARG00020020746 | *PSMA3* | -0.332 | 0.10227 |
| ENSOARG00020003892 | *SELENOS* | -0.404 | 0.10284 |
| ENSOARG00020015439 | *KIF1B* | 0.459 | 0.10362 |
| ENSOARG00020016933 | *IDH1* | -0.436 | 0.10362 |
| ENSOARG00020014594 | *CBR1* | -0.880 | 0.10362 |
| ENSOARG00020009901 |  | -0.346 | 0.10363 |
| ENSOARG00020000694 | *TEX10* | -0.358 | 0.10420 |
| ENSOARG00020016167 | *CUX2* | -0.643 | 0.10448 |
| ENSOARG00020016159 | *TCP11L2* | 0.695 | 0.10462 |
| ENSOARG00020023960 | *NDUFB8* | -0.277 | 0.10462 |
| ENSOARG00020002750 | *SLC6A9* | 0.889 | 0.10481 |
| ENSOARG00020007434 |  | 0.603 | 0.10507 |
| ENSOARG00020024877 |  | 0.483 | 0.10570 |
| ENSOARG00020004052 | *MRPS31* | -0.411 | 0.10570 |
| ENSOARG00020001868 | *USP20* | 0.397 | 0.10585 |
| ENSOARG00020023031 | *CCDC186* | 0.383 | 0.10585 |
| ENSOARG00020005063 | *CLCC1* | 0.351 | 0.10585 |
| ENSOARG00020022123 | *BANF1* | -0.335 | 0.10585 |
| ENSOARG00020008915 | *SWI5* | -0.354 | 0.10585 |
| ENSOARG00020025228 | *PIN4* | -0.544 | 0.10585 |
| ENSOARG00020000286 | *RTP4* | -0.561 | 0.10585 |
| ENSOARG00020026084 | *NUPR1* | -0.595 | 0.10585 |
| ENSOARG00020000127 | *PTGR1* | -0.610 | 0.10585 |
| ENSOARG00020011887 |  | -0.433 | 0.10631 |
| ENSOARG00020001706 | *RXRA* | 0.228 | 0.10659 |
| ENSOARG00020003296 | *RASAL3* | -0.680 | 0.10659 |
| ENSOARG00020007348 | *OTUD7B* | 0.384 | 0.10709 |
| ENSOARG00020019197 | *DENND1C* | -0.385 | 0.10815 |
| ENSOARG00020017617 | *ACSL5* | -0.449 | 0.10815 |
| ENSOARG00020005611 | *ALG5* | -0.336 | 0.10833 |
| ENSOARG00020025653 | *NT5DC2* | 0.513 | 0.10846 |
| ENSOARG00020013605 | *NUP210* | 0.407 | 0.10846 |
| ENSOARG00020000212 | *TOMM40L* | -0.347 | 0.10846 |
| ENSOARG00020008550 | *SNRPF* | -0.373 | 0.10846 |
| ENSOARG00020013027 | *NDUFA7* | -0.439 | 0.10846 |
| ENSOARG00020012417 | *BHLHE40* | -0.546 | 0.10846 |
| ENSOARG00020012777 | *DDX20* | 0.306 | 0.10944 |
| ENSOARG00020008933 | *MIX23* | -0.442 | 0.10944 |
| ENSOARG00020024196 | *TMA7* | -0.299 | 0.10945 |
| ENSOARG00020001613 | *ATOSB* | 0.496 | 0.11022 |
| ENSOARG00020024983 | *SEC11C* | -0.516 | 0.11071 |
| ENSOARG00020006701 | *LGALS1* | -0.837 | 0.11071 |
| ENSOARG00020025397 | *LDB1* | 0.359 | 0.11300 |
| ENSOARG00020010173 | *MON2* | 0.242 | 0.11300 |
| ENSOARG00020024501 |  | -0.305 | 0.11300 |
| ENSOARG00020024215 | *MFHAS1* | 0.293 | 0.11477 |
| ENSOARG00020025986 | *SDK2* | 0.555 | 0.11544 |
| ENSOARG00020018871 |  | -0.336 | 0.11544 |
| ENSOARG00020001922 | *ZC3H8* | -0.397 | 0.11548 |
| ENSOARG00020022029 | *PDZD11* | -0.299 | 0.11605 |
| ENSOARG00020025448 | *MRPL15* | -0.342 | 0.11632 |
| ENSOARG00020009469 | *NOP16* | -0.450 | 0.11766 |
| ENSOARG00020011475 | *ANXA9* | 0.882 | 0.11782 |
| ENSOARG00020024417 | *SLC29A1* | 0.378 | 0.11826 |
| ENSOARG00020020936 | *PIK3R5* | 0.874 | 0.11902 |
| ENSOARG00020020785 |  | -0.859 | 0.11902 |
| ENSOARG00020005533 |  | 0.392 | 0.11902 |
| ENSOARG00020020089 | *TM6SF2* | -0.521 | 0.11902 |
| ENSOARG00020009646 | *SLC7A1* | 0.803 | 0.11942 |
| ENSOARG00020019774 | *ACCS* | -0.322 | 0.11942 |
| ENSOARG00020001464 | *FKBP11* | -0.465 | 0.11942 |
| ENSOARG00020001817 |  | -0.792 | 0.11960 |
| ENSOARG00020025852 | *ALAS1* | -0.463 | 0.11960 |
| ENSOARG00020012944 | *GCHFR* | -0.332 | 0.11999 |
| ENSOARG00020012231 | *RTN4IP1* | -0.415 | 0.12054 |
| ENSOARG00020009410 | *MCM3AP* | 0.234 | 0.12127 |
| ENSOARG00020026442 |  | -0.380 | 0.12127 |
| ENSOARG00020009496 | *PET100* | -0.464 | 0.12127 |
| ENSOARG00020021909 | *DSTN* | -0.471 | 0.12127 |
| ENSOARG00020019585 | *DCUN1D5* | -0.473 | 0.12127 |
| ENSOARG00020007895 | *ORC1* | 1.053 | 0.12146 |
| ENSOARG00020003931 | *ATP2B4* | 0.638 | 0.12146 |
| ENSOARG00020003478 | *DDX56* | -0.244 | 0.12146 |
| ENSOARG00020022449 | *MTRR* | -0.348 | 0.12146 |
| ENSOARG00020009684 |  | 0.453 | 0.12146 |
| ENSOARG00020026234 | *PEMT* | -0.390 | 0.12146 |
| ENSOARG00020001480 | *ASB1* | 0.472 | 0.12253 |
| ENSOARG00020004400 | *SPCS2* | -0.293 | 0.12253 |
| ENSOARG00020018721 | *ANKS4B* | -0.301 | 0.12253 |
| ENSOARG00020001080 |  | -0.660 | 0.12253 |
| ENSOARG00020012161 | *C1orf131* | -0.450 | 0.12253 |
| ENSOARG00020004084 | *ICAM5* | 1.296 | 0.12385 |
| ENSOARG00020010196 | *NOB1* | -0.267 | 0.12418 |
| ENSOARG00020014856 | *GPN3* | -0.326 | 0.12418 |
| ENSOARG00020026346 |  | -0.233 | 0.12418 |
| ENSOARG00020010547 | *FUT10* | -0.394 | 0.12418 |
| ENSOARG00020017036 |  | -0.428 | 0.12471 |
| ENSOARG00020000483 | *CPOX* | 0.806 | 0.12486 |
| ENSOARG00020001942 | *TFRC* | 0.800 | 0.12486 |
| ENSOARG00020005456 | *CALU* | -0.291 | 0.12486 |
| ENSOARG00020003246 | *NDUFS4* | -0.292 | 0.12486 |
| ENSOARG00020006056 | *ZNF131* | -0.308 | 0.12486 |
| ENSOARG00020008052 | *POLR2F* | -0.318 | 0.12486 |
| ENSOARG00020020001 | *PARK7* | -0.330 | 0.12486 |
| ENSOARG00020002174 | *RAB17* | -0.366 | 0.12486 |
| ENSOARG00020019330 |  | -0.861 | 0.12486 |
| ENSOARG00020020520 |  | 0.462 | 0.12486 |
| ENSOARG00020001092 | *ALG14* | -0.428 | 0.12486 |
| ENSOARG00020024805 | *CCDC134* | -0.477 | 0.12486 |
| ENSOARG00020002706 | *MX1* | -0.607 | 0.12486 |
| ENSOARG00020007692 | *AKR7L* | 0.627 | 0.12524 |
| ENSOARG00020005097 | *KLHL24* | 0.553 | 0.12606 |
| ENSOARG00020005744 | *CWC27* | -0.260 | 0.12606 |
| ENSOARG00020021395 | *MAP3K6* | 0.473 | 0.12628 |
| ENSOARG00020022113 | *DNMT3A* | 0.257 | 0.12628 |
| ENSOARG00020001624 | *NANS* | -0.563 | 0.12628 |
| ENSOARG00020004202 | *TONSL* | 0.458 | 0.12667 |
| ENSOARG00020006402 | *TXNRD2* | 0.438 | 0.12667 |
| ENSOARG00020013642 | *FBXO25* | 0.317 | 0.12667 |
| ENSOARG00020007295 | *SH3BGRL3* | -0.313 | 0.12667 |
| ENSOARG00020009071 | *NDUFB5* | -0.412 | 0.12667 |
| ENSOARG00020004073 |  | 0.726 | 0.12667 |
| ENSOARG00020020886 | *NTN1* | 0.511 | 0.12670 |
| ENSOARG00020018988 | *WDFY1* | 0.394 | 0.12670 |
| ENSOARG00020023888 | *VTN* | -0.438 | 0.12670 |
| ENSOARG00020004415 | *PLEKHA6* | -0.267 | 0.12696 |
| ENSOARG00020026191 | *NME1* | -0.276 | 0.12696 |
| ENSOARG00020000828 | *CYB5R3* | -0.245 | 0.12762 |
| ENSOARG00020026121 | *MPC1* | -0.334 | 0.12762 |
| ENSOARG00020015215 | *SELENBP1* | -0.632 | 0.12762 |
| ENSOARG00020006131 | *CCDC124* | -0.229 | 0.12790 |
| ENSOARG00020006221 | *DENND4A* | 0.578 | 0.12817 |
| ENSOARG00020003471 | *SLC38A2* | 0.537 | 0.12867 |
| ENSOARG00020011626 | *CBX6* | 0.491 | 0.12867 |
| ENSOARG00020002745 | *HDAC7* | 0.310 | 0.12867 |
| ENSOARG00020000683 | *CEP41* | -0.313 | 0.12867 |
| ENSOARG00020025436 | *EXOSC7* | -0.388 | 0.12867 |
| ENSOARG00020002211 | *ADAMTSL2* | -0.526 | 0.12867 |
| ENSOARG00020023087 | *FAH* | -0.340 | 0.12923 |
| ENSOARG00020021882 | *DENND4C* | 0.371 | 0.12962 |
| ENSOARG00020012783 | *NFATC1* | 0.342 | 0.12962 |
| ENSOARG00020008033 | *NEIL2* | -0.390 | 0.12962 |
| ENSOARG00020017395 | *LZIC* | -0.370 | 0.12969 |
| ENSOARG00020023382 | *MORF4L2* | -0.196 | 0.12976 |
| ENSOARG00020011633 | *MRPL34* | -0.304 | 0.12976 |
| ENSOARG00020016663 | *FGG* | -0.340 | 0.12976 |
| ENSOARG00020003775 | *TMEM242* | -0.475 | 0.12976 |
| ENSOARG00020009382 | *WASHC3* | -0.313 | 0.13075 |
| ENSOARG00020005373 | *TLN1* | 0.300 | 0.13250 |
| ENSOARG00020004954 | *CUBN* | 2.179 | 0.13300 |
| ENSOARG00020007076 | *CARM1* | 0.320 | 0.13300 |
| ENSOARG00020021462 | *TMEM223* | -0.369 | 0.13300 |
| ENSOARG00020012719 | *BROX* | 0.267 | 0.13335 |
| ENSOARG00020019301 | *RPL26L1* | -0.393 | 0.13335 |
| ENSOARG00020009606 | *SMCHD1* | 0.426 | 0.13369 |
| ENSOARG00020011039 | *DGKQ* | -0.390 | 0.13386 |
| ENSOARG00020003183 | *TBC1D13* | 0.282 | 0.13390 |
| ENSOARG00020019868 | *DPH7* | -0.307 | 0.13418 |
| ENSOARG00020001842 | *AUP1* | -0.320 | 0.13418 |
| ENSOARG00020017215 | *PIGK* | -0.294 | 0.13423 |
| ENSOARG00020000918 | *EML6* | 0.985 | 0.13433 |
| ENSOARG00020026384 | *EPN2* | 0.286 | 0.13433 |
| ENSOARG00020018319 | *CDAN1* | 0.280 | 0.13433 |
| ENSOARG00020002085 | *SNX17* | -0.196 | 0.13433 |
| ENSOARG00020024579 | *PMM1* | -0.266 | 0.13433 |
| ENSOARG00020022847 | *YIF1A* | -0.286 | 0.13433 |
| ENSOARG00020005662 | *ENTPD6* | -0.411 | 0.13433 |
| ENSOARG00020019431 | *CREBRF* | 0.402 | 0.13646 |
| ENSOARG00020021193 | *RPLP0* | 0.311 | 0.13661 |
| ENSOARG00020022801 | *MXD4* | 0.558 | 0.13683 |
| ENSOARG00020019506 | *FBXO7* | 0.484 | 0.13683 |
| ENSOARG00020010094 | *APPBP2* | 0.404 | 0.13683 |
| ENSOARG00020008984 | *CHAMP1* | 0.268 | 0.13683 |
| ENSOARG00020016433 | *NCKAP1* | 0.240 | 0.13683 |
| ENSOARG00020015244 | *IQSEC1* | 0.219 | 0.13683 |
| ENSOARG00020018378 | *ACOX3* | -0.189 | 0.13683 |
| ENSOARG00020021339 | *CDK5RAP3* | -0.232 | 0.13683 |
| ENSOARG00020008781 | *GMPR2* | -0.257 | 0.13683 |
| ENSOARG00020009062 | *HSD17B10* | -0.307 | 0.13683 |
| ENSOARG00020003046 | *SHFL* | -0.308 | 0.13683 |
| ENSOARG00020003490 | *NOP10* | -0.327 | 0.13683 |
| ENSOARG00020002131 | *BZW2* | -0.342 | 0.13683 |
| ENSOARG00020019649 | *YARS1* | -0.349 | 0.13683 |
| ENSOARG00020009112 | *H1-3* | -0.392 | 0.13683 |
| ENSOARG00020025442 | *INCA1* | -0.542 | 0.13683 |
| ENSOARG00020023310 |  | 0.417 | 0.13683 |
| ENSOARG00020025619 |  | 1.038 | 0.13683 |
| ENSOARG00020007751 | *NT5C2* | -0.297 | 0.13683 |
| ENSOARG00020002089 |  | -0.894 | 0.13683 |
| ENSOARG00020005487 |  | -0.348 | 0.13683 |
| ENSOARG00020026086 | *CRKL* | 0.205 | 0.13722 |
| ENSOARG00020004685 | *GNPNAT1* | -0.328 | 0.13722 |
| ENSOARG00020008195 | *TRMT112* | -0.335 | 0.13722 |
| ENSOARG00020016569 | *AGXT2* | -0.391 | 0.13722 |
| ENSOARG00020003097 | *SMARCC2* | 0.172 | 0.13734 |
| ENSOARG00020012900 | *WDR83OS* | -0.268 | 0.13734 |
| ENSOARG00020008066 | *KL* | 0.896 | 0.13741 |
| ENSOARG00020022281 | *HNF1B* | -0.453 | 0.13831 |
| ENSOARG00020022416 | *MAP4K5* | 0.469 | 0.13968 |
| ENSOARG00020013929 | *SH3RF1* | 0.366 | 0.14009 |
| ENSOARG00020017795 | *ELP5* | -0.242 | 0.14087 |
| ENSOARG00020001351 | *RFX1* | 0.272 | 0.14107 |
| ENSOARG00020010032 | *TGFBI* | -0.349 | 0.14139 |
| ENSOARG00020023642 | *AGT* | -0.494 | 0.14140 |
| ENSOARG00020003833 | *NDUFA5* | -0.368 | 0.14143 |
| ENSOARG00020010715 | *FUZ* | -0.373 | 0.14173 |
| ENSOARG00020011786 |  | -0.431 | 0.14196 |
| ENSOARG00020008510 | *CARMIL1* | 0.362 | 0.14388 |
| ENSOARG00020019993 | *SUGP1* | -0.232 | 0.14388 |
| ENSOARG00020015489 | *TOMM6* | -0.336 | 0.14388 |
| ENSOARG00020016698 | *UBR2* | 0.297 | 0.14421 |
| ENSOARG00020010229 | *INTS11* | -0.241 | 0.14486 |
| ENSOARG00020018089 | *NEK9* | -0.291 | 0.14486 |
| ENSOARG00020006229 | *HSBP1* | -0.412 | 0.14486 |
| ENSOARG00020021181 | *FBXO9* | 0.504 | 0.14528 |
| ENSOARG00020012835 | *WDR83* | -0.370 | 0.14528 |
| ENSOARG00020003333 | *MOCS2* | -0.395 | 0.14528 |
| ENSOARG00020018202 | *TSC22D4* | 0.281 | 0.14547 |
| ENSOARG00020010527 | *AP2A1* | 0.492 | 0.14557 |
| ENSOARG00020006416 | *FCHO1* | 0.439 | 0.14610 |
| ENSOARG00020020336 | *URB2* | 0.342 | 0.14610 |
| ENSOARG00020018298 | *PUM2* | 0.272 | 0.14610 |
| ENSOARG00020001839 | *SEC62* | -0.231 | 0.14610 |
| ENSOARG00020020260 | *PLA2G12B* | -0.245 | 0.14610 |
| ENSOARG00020020899 | *PFDN1* | -0.259 | 0.14610 |
| ENSOARG00020019082 | *CDIPT* | -0.266 | 0.14610 |
| ENSOARG00020004890 | *SPP2* | -0.456 | 0.14610 |
| ENSOARG00020004363 | *ITIH5* | -0.723 | 0.14610 |
| ENSOARG00020021749 | *PYROXD2* | -0.877 | 0.14610 |
| ENSOARG00020006483 |  | -0.608 | 0.14767 |
| ENSOARG00020002804 | *SMC2* | 0.847 | 0.14788 |
| ENSOARG00020019362 | *ATP6V0E1* | -0.255 | 0.14790 |
| ENSOARG00020018336 | *RPL12* | 0.252 | 0.14791 |
| ENSOARG00020022178 | *ENTPD8* | -0.353 | 0.14791 |
| ENSOARG00020005170 | *TTC7A* | 0.415 | 0.14830 |
| ENSOARG00020020285 | *AMT* | -0.225 | 0.14830 |
| ENSOARG00020010477 | *ENTREP3* | -0.308 | 0.14830 |
| ENSOARG00020019545 | *SRPRB* | -0.311 | 0.14830 |
| ENSOARG00020009135 | *MRPL17* | -0.394 | 0.14830 |
| ENSOARG00020005732 | *ALG12* | -0.506 | 0.14830 |
| ENSOARG00020011795 | *CRLF3* | 0.721 | 0.14862 |
| ENSOARG00020021528 | *RFTN1* | 0.386 | 0.14862 |
| ENSOARG00020026360 | *SMCR8* | 0.377 | 0.14862 |
| ENSOARG00020009753 |  | -0.436 | 0.14862 |
| ENSOARG00020010254 | *PPM1D* | 0.457 | 0.14910 |
| ENSOARG00020019929 | *MEGF8* | 0.273 | 0.14910 |
| ENSOARG00020008122 |  | -0.299 | 0.14910 |
| ENSOARG00020012982 | *GRK6* | 0.385 | 0.14990 |
| ENSOARG00020020927 | *FAM222B* | 0.364 | 0.14990 |
| ENSOARG00020020554 | *EEF1A1* | 0.203 | 0.14990 |
| ENSOARG00020000902 | *S100A14* | -0.376 | 0.14990 |
| ENSOARG00020006358 |  | -0.621 | 0.14990 |

**Table S3.** Canonical pathways predicted using differentially expressed genes regulated by hypoxia in fetal livers.

| Canonical pathway name | -Log(*P*) | *Z*-score | Predicted activation |
| --- | --- | --- | --- |
| Granzyme A Signaling | 4.86 | 2.53 | Up-regulated |
| Metabolism of water-soluble vitamins & cofactors | 4.57 | -2.333 | Down-regulated |
| SRP-dependent cotranslational protein targeting to membrane | 4.54 | -2.309 | Down-regulated |
| Mitochondrial translation | 3.78 | -3.162 | Down-regulated |
| rRNA processing | 3.48 | N/A |  |
| Mitochondrial Dysfunction | 3.37 | 3.578 | Up-regulated |
| Oxidative Phosphorylation | 3.37 | -3.162 | Down-regulated |
| Asparagine N-linked glycosylation | 2.9 | -2.236 | Down-regulated |
| Electron transport & ATP synthesis by uncoupling proteins | 2.78 | -3.162 | Down-regulated |
| Ascorbate Recycling (Cytosolic) | 2.65 | N/A |  |
| Metabolism of non-coding RNA | 2.58 | 0 |  |
| BMAL1:CLOCK,NPAS2 activates circadian gene expression | 2.34 | 0 |  |
| Colonic Acid Building Blocks Biosynthesis | 2.22 | N/A |  |
| Nucleotide catabolism | 2.22 | -2 | Down-regulated |
| Sirtuin Signaling Pathway | 2.11 | 1.897 | Up-regulated |
| Hematoma Resolution Signaling Pathway | 2 | -2.496 | Down-regulated |
| ISG15 antiviral mechanism | 1.99 | -0.816 | Down-regulated |
| Glycine Cleavage Complex | 1.98 | N/A |  |
| GDP-mannose Biosynthesis | 1.98 | N/A |  |
| Cellular response to heat stress | 1.82 | -1 | Down-regulated |
| Detoxification of Reactive Oxygen Species | 1.78 | -1 | Down-regulated |
| Serine biosynthesis | 1.72 | N/A |  |
| Chaperone Mediated Autophagy | 1.72 | N/A |  |
| Vitamin-C Transport | 1.72 | N/A |  |
| Interconversion of nucleotide di- and triphosphates | 1.66 | N/A |  |
| Pyrimidine Ribonucleotides De Novo Biosynthesis | 1.66 | -2 | Down-regulated |
| Post-translational protein phosphorylation | 1.63 | -1.89 | Down-regulated |
| Heme Biosynthesis II | 1.62 | N/A |  |
| Tryptophan Degradation III (Eukaryotic) | 1.61 | N/A |  |
| DHCR24 Signaling Pathway | 1.6 | -1.414 | Down-regulated |
| Adenine and Adenosine Salvage VI | 1.56 | N/A |  |
| D-mannose Degradation | 1.56 | N/A |  |
| Neutrophil Extracellular Trap Signaling Pathway | 1.56 | -1.807 | Down-regulated |
| Dolichyl-diphosphooligosaccharide Biosynthesis | 1.53 | N/A |  |
| Signaling by Erythropoietin | 1.52 | N/A |  |
| Phase II - Conjugation of compounds | 1.46 | -1 | Down-regulated |
| Complement cascade | 1.46 | -2 | Down-regulated |
| Phenylalanine and tyrosine metabolism | 1.45 | N/A |  |
| Interferon Signaling | 1.43 | N/A |  |
| Glucocorticoid Receptor Signaling | 1.4 | N/A |  |
| Regulation of IGF transport & uptake by IGFBPs | 1.38 | -1.89 | Down-regulated |
| Advanced glycosylation endproduct receptor signaling | 1.38 | N/A |  |
| Mitotic Prophase | 1.36 | 0 |  |
| TP53 Regulates Transcription of Cell Cycle Genes | 1.34 | 1 | Up-regulated |

^a^Listed in order of *P*-value.

^b^Positive Z-score indicates predicted pathway up-regulation and negative scores indicate predicted down-regulation. A N/A z-score indicates that IPA software did not predict a score and did predict a pathway activation state.

**Table S4.** Predicted upstream regulators.

| Upstream regulator | -Log(*P*) | *Z*-score | Predicted activation |
| --- | --- | --- | --- |
| HNF4A | 6.167 | -1.809 |  |
| XBP1 | 5.426 | -4.14 | Inhibited |
| KDM5A | 4.234 | 3.5 | Activated |
| STK17A | 3.402 | N/A |  |
| HBA1/HBA2 | 3.311 | -2.449 | Inhibited |
| LONP1 | 2.967 | -1.067 |  |
| CAB39L | 2.701 | -2.236 | Inhibited |
| EPO | 2.670 | 1.291 |  |
| TAOK2 | 2.648 | N/A |  |
| KRAS | 2.636 | 1.835 |  |
| ELAC2 | 2.530 | N/A |  |
| HTT | 2.441 | N/A |  |
| CLUH | 2.437 | 0.954 |  |
| UCP1 | 2.381 | -2.198 | Inhibited |
| MAP4K4 | 2.356 | 3 | Activated |
| DOHH | 2.355 | N/A |  |
| PAPOLA | 2.355 | N/A |  |
| IFNL1 | 2.281 | -2.38 | Inhibited |
| NKX2-3 | 2.277 | 1.838 |  |
| Hbb-b1 | 2.236 | -2.449 | Inhibited |
| RNF187 | 2.236 | 1.219 |  |
| ABCC8 | 2.213 | N/A |  |
| ERN1 | 2.208 | -2.538 | Inhibited |
| PSEN1 | 2.208 | 0.132 |  |
| RB1 | 2.167 | -3.333 | Inhibited |
| GAPDH | 2.148 | N/A |  |
| FBXL5 | 2.140 | N/A |  |
| SIRT3 | 2.140 | 0.397 |  |
| SDHB | 2.126 | N/A |  |
| ATF4 | 2.100 | -0.664 |  |
| IRF2 | 2.082 | -0.6 |  |
| PRKAG3 | 2.027 | N/A |  |
| FOS | 1.975 | 0.45 |  |
| PFDN5 | 1.971 | N/A |  |
| PGRMC2 | 1.971 | N/A |  |
| SOD1 | 1.963 | N/A |  |
| PNPT1 | 1.947 | 2 | Activated |
| PHF12 | 1.947 | 2 | Activated |
| SMYD1 | 1.917 | N/A |  |
| NSUN3 | 1.833 | N/A |  |
| DHPS | 1.833 | N/A |  |
| NPAS2 | 1.833 | N/A |  |
| SLC29A1 | 1.833 | N/A |  |
| SNCA | 1.830 | -0.577 |  |
| ACOX1 | 1.812 | -0.113 |  |
| EIF2AK2 | 1.780 | -2.393 | Inhibited |
| TWNK | 1.772 | N/A |  |
| AURKB | 1.772 | N/A |  |
| VDR | 1.767 | 0.63 |  |
| F2 | 1.740 | -1.471 |  |
| TXNRD1 | 1.730 | N/A |  |
| NQO1 | 1.688 | -2.213 | Inhibited |
| SREBF1 | 1.688 | -3.046 | Inhibited |
| GPX1 | 1.654 | N/A |  |
| TNFSF11 | 1.650 | -0.585 |  |
| HIBCH | 1.629 | 1.342 |  |
| DYRK1B | 1.629 | 1.274 |  |
| NFE2L2 | 1.625 | -1.668 |  |
| FAN1 | 1.616 | N/A |  |
| MPST | 1.616 | N/A |  |
| SKIL | 1.616 | 1.982 |  |
| PPP3CA | 1.583 | N/A |  |
| COX10 | 1.558 | N/A |  |
| GPLD1 | 1.558 | N/A |  |
| RGS5 | 1.558 | N/A |  |
| MPP3 | 1.558 | N/A |  |
| TAOK1 | 1.558 | N/A |  |
| ACE | 1.558 | N/A |  |
| NUDT1 | 1.558 | N/A |  |
| ASB9 | 1.558 | N/A |  |
| ZNF667 | 1.558 | N/A |  |
| NKX2-8 | 1.558 | N/A |  |
| LIMD1 | 1.558 | N/A |  |
| Hsf4 | 1.558 | N/A |  |
| SLC31A1 | 1.558 | N/A |  |
| PRELID1 | 1.558 | N/A |  |
| AP2S1 | 1.558 | N/A |  |
| TMED10 | 1.558 | N/A |  |
| NONO | 1.554 | -2.621 | Inhibited |
| IFNA10 | 1.527 | N/A |  |
| IFNA21 | 1.527 | N/A |  |
| IFNA5 | 1.527 | N/A |  |
| IFNA7 | 1.527 | N/A |  |
| IFNA6 | 1.527 | N/A |  |
| IFNL4 | 1.527 | N/A |  |
| XRCC6 | 1.527 | N/A |  |
| FXN | 1.527 | N/A |  |
| ATXN3 | 1.527 | N/A |  |
| NACC1 | 1.527 | N/A |  |
| HELLS | 1.521 | 1.342 |  |
| RBM39 | 1.510 | N/A |  |
| TRIM24 | 1.495 | 2.236 | Activated |
| AGMAT | 1.466 | N/A |  |
| IFNA8 | 1.447 | N/A |  |
| IFNA16 | 1.447 | N/A |  |
| CYP46A1 | 1.447 | N/A |  |
| LOC102724428/SIK1 | 1.447 | N/A |  |
| DDB2 | 1.447 | N/A |  |
| PIAS3 | 1.447 | N/A |  |
| ATRX | 1.447 | N/A |  |
| ALDH2 | 1.424 | N/A |  |
| CNOT7 | 1.424 | N/A |  |
| TREX1 | 1.407 | 2.155 | Activated |
| GSR | 1.383 | N/A |  |
| SUB1 | 1.383 | N/A |  |
| CAPN3 | 1.377 | N/A |  |
| TFRC | 1.356 | -2.646 | Inhibited |
| PAX6 | 1.353 | 0.218 |  |
| SLC13A1 | 1.352 | 0.447 |  |
| MYCN | 1.338 | 0.75 |  |
| CLPP | 1.330 | 2.213 | Activated |
| PTPRR | 1.316 | 0.816 |  |
| MYO1C | 1.312 | N/A |  |
| SLC40A1 | 1.312 | N/A |  |
| ETV6 | 1.308 | 1.951 |  |

^a^Listed in order of p value.

^b^Positive Z-score indicates predicted activated upstream regulator pathways and negative scores indicate predicted inhibited regulator pathways. A N/A z-score indicates that IPA software did not predict a score and did predict a pathway activation state.

**Table S5.** Predicted metabolic pathways enriched in differentially expressed genes and top 40 metabolites with the highest-ranking VIP scores.

| Pathway | -Log(*P*) | Enrichment ratio |
| --- | --- | --- |
| Protein export | 4.124 | 10.916 |
| Glycine, serine and threonine metabolism | 3.380 | 4.463 |
| Ribosome | 3.016 | 3.282 |
| N-Glycan biosynthesis | 2.737 | 3.994 |
| Protein processing in endoplasmic reticulum | 2.634 | 2.919 |
| Endocytosis | 2.296 | 2.363 |
| Huntington disease | 2.231 | 2.562 |
| Mineral absorption | 2.113 | 3.463 |
| Oxidative phosphorylation | 2.005 | 2.696 |
| Glycerophospholipid metabolism | 2.005 | 2.696 |
| Non-alcoholic fatty liver disease (NAFLD) | 1.973 | 2.660 |
| Glutathione metabolism | 1.955 | 3.205 |
| Thermogenesis | 1.891 | 2.175 |
| Parkinson disease | 1.878 | 2.559 |
| Purine metabolism | 1.834 | 2.232 |
| Ribosome biogenesis in eukaryotes | 1.736 | 2.869 |
| Central carbon metabolism in cancer | 1.718 | 2.842 |
| RNA transport | 1.717 | 2.391 |
| Complement and coagulation cascades | 1.667 | 3.138 |
| Cysteine and methionine metabolism | 1.613 | 2.690 |
| Peroxisome | 1.605 | 3.025 |
| Alzheimer disease | 1.584 | 2.257 |
| Vitamin B6 metabolism | 1.528 | 4.431 |
| Synaptic vesicle cycle | 1.473 | 2.790 |
| Pyrimidine metabolism | 1.455 | 2.469 |
| Amino sugar and nucleotide sugar metabolism | 1.438 | 2.253 |
| Glyoxylate and dicarboxylate metabolism | 1.438 | 2.729 |
| Glycosylphosphatidylinositol (GPI)-anchor biosynthesis | 1.375 | 3.862 |
| Phenylalanine, tyrosine and tryptophan biosynthesis | 1.348 | 3.766 |
